# Rapid and supersensitive allele detection of *Plasmodium falciparum* chloroquine resistance via a *Pyrococcus furiosus* argonaute-triggered dual-signal biosensing platform

**DOI:** 10.1186/s13071-024-06575-0

**Published:** 2024-11-24

**Authors:** Liying Chen, Wencheng Chen, Huagui Wei, Wenai Lin, Cheng Zhang, Hongfei Hu, Chunfang Wang, Jiangtao Chen, Xueyan Liang, Daiqian Zhu, Junli Wang, Zongyun Lin, Yuxia Wei, Jian Li, Min Lin

**Affiliations:** 1 Department of Reproductive Medicine, Affiliated Hospital of Youjiang Medical University for Nationalities, Baise, Guangxi China; 2 School of Basic Medicine Science, Hubei University of Medicine, Shiyan, Hubei China; 3 Laboratory Medical Center, Affiliated Hospital of Youjiang Medical University for Nationalities, Baise, Guangxi China; 4 Modern Industrial College of Biomedicine and Great Health, Youjiang Medical University for Nationalities, Baise, Guangxi China; 5 Guangxi Medical and Health Key Discipline Construction Project of the Affiliated Hospital of Youjiang Medical University for Nationalities, Baise, Guangxi China; 6 Key Laboratory of Research On Clinical Molecular Diagnosis for High Incidence Diseases in Western Guangxi of Guangxi Higher Education Institutions, Baise, Guangxi China; 7 Laboratory Medical Center, Huizhou Municipal Central Hospital, Huizhou, Guangdong China

**Keywords:** *Pyrococcus furiosus* argonaute (*Pf*Ago), Recombinase polymerase amplification (RPA), *Plasmodium falciparum* chloroquine resistance transporter (*Pfcrt*), Genotyping, Haplotype detection, Point-of-care testing (POCT)

## Abstract

**Background:**

Malaria remains a serious public health problem worldwide, particularly in Africa. Resistance to antimalarial drugs is an essential issue for malaria control and elimination. Currently, polymerase chain reaction (PCR) combined with Sanger sequencing is regarded as the gold standard for mutation detection. However, this method fails to meet the requirements of point-of-care testing (POCT) because of its time-consuming, expensive instruments and professional dependence. To support this strategy, we developed a novel diagnostic platform that combines recombinase polymerase amplification (RPA) with the *Pyrococcus furiosus* argonaute (*Pf*Ago) protein and was designed to detect gene mutations related to antimalarial drug resistance. The *Pfcrt* haplotypes CVMNK and CVIET of chloroquine resistance (CQR) were used as examples and were assessed in this study.

**Methods:**

By meticulously designing strategies, RPA primers, guide DNAs, and probes were screened, the reaction was optimized, and the resulting parameters were employed to ascertain the genotype of *Pfcrt*. The recombinant plasmids *pUC57/Pfcrt*-CVIET and *pUC57/Pfcrt*-CVMNK were constructed and diluted for sensitivity detection. The *pUC57/Pfcrt*-CVIET plasmid mixture was added to the *pUC57/Pfcrt*-CVMNK plasmid mixture in different additions to configure several specific proportions of mixed plasmid mixtures. The RPA-*Pf*Ago platform was used, and the mixed plasmid was detected simultaneously via nest-PCR (nPCR) and Sanger sequencing. The platform was then evaluated on 85 clinical samples and compared with Sanger sequencing.

**Results:**

The entire process achieves the key mutation *Pfcrt*-CVMNK/CVIET genotype identification of CQR within 90 min. The platform achieved 1.8 × 10^4^ copies/μL sensitivity and could detect as little as 3% CVIET in mixed plasmids, which is a higher sensitivity than that of Sanger sequencing (5%). Notably, the platform shows 100% concordance with the gold standard method when 85 clinical samples are tested. The sensitivity and specificity were 100% for the 85 clinical samples.

**Conclusions:**

This study established an RPA-*Pf*Ago platform for genotyping the key mutation *Pfcrt*-CVMNK/CVIET of CQR. This method can rapidly produce reliable results and avoid the disadvantages of nPCR with sequencing. This approach has the characteristics of a short operation time, low device dependence, and a good match to the POCT strategy, suggesting that the platform can be easily applied locally or on site.

**Graphical abstract:**

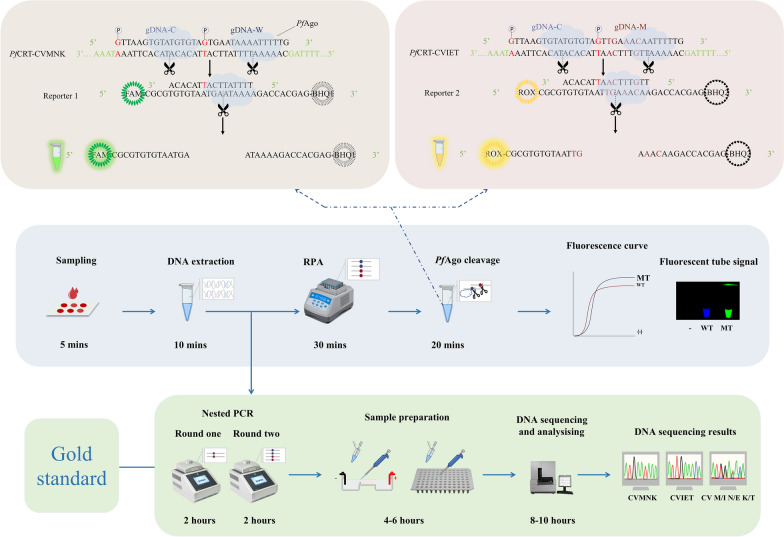

**Supplementary Information:**

The online version contains supplementary material available at 10.1186/s13071-024-06575-0.

## Background

Malaria is recognized as a major public problem worldwide, and it is estimated that more than 600,000 individuals will die annually [1]. Most of them are centralized in resource-poor countries and areas, particularly in sub-Saharan Africa and Southeast Asia [2]. For malaria control, treatment with effective, safe and affordable antimalarial drugs is largely needed. The emergence and rapid spread of drug resistance have severely compromised this strategy and have been linked to increases in malaria-associated morbidity and mortality. Chloroquine (CQ), the least expensive basic antimalarial drug, has lost its efficacy in most parts of Africa [3]. *Pfcrt* haplotypes associated with CQ resistance (CQR) in natural parasite isolates harbor threonine (T), as opposed to lysine (K), at amino acid 76 *P*. *falciparum* CQR strains carrying the CVIET haplotype (residues 72–76), and chloroquine-sensitive strains with the CVMNK haplotype are commonly detected in Africa. In contrast, CQR strains with the SVMNT haplotype are rarely detected in Africa [4]. Accurate confirmation of these single-nucleotide polymorphism (SNP) loci and alleles will aid in medication guidance [5, 6].

Nest-PCR (nPCR) and Sanger sequencing are considered the recommended methods for identifying *Plasmodium* parasite mutations related to antimalarial drug resistance [7]. Compared with authentication by sequencing DNA fragments, PCR-restriction fragment length polymorphism (PCR–RFLP), allele-specific diagnostic PCR (AS-PCR) [2, 8], and real-time PCR are practical and effective, but they require sophisticated thermal cyclers with trained personnel, which makes their use difficult in underequipped laboratories and low-resource field settings [7, 9, 10]. Thus, they fail to meet the requirements of the point-of-care testing strategy.

To overcome these limitations, new alternative genotyping methods for key mutations, especially isothermal amplification technologies combined with programmable endonuclease-based methods, have been developed [11–14]. Furthermore, prokaryotic argonaute is the key protein in the host defense system that functions by mediating nucleic acid molecules [15–17]. As a DNA-guided endonuclease from *Pyrococcus furiosus*, the *Pyrococcus furiosus* argonaute (*Pf*Ago) prefers to cut target cognate DNA under the guidance of short 5′-phosphorylated single-strand DNA without the need for a protospacer-adjacent motif (PAM) or PFS (protospacer flanking site) in the target sequence, which largely extends its application in the selection of available target DNA sequences [18, 19]. Recently, it has been applied to the molecular detection of the key SARS-CoV-2 mutation L452R [14], which was identified in delta and omicorn BA.5 variants [20, 21]. However, *Pf*Ago-based technology has yet to be applied to identify the genotypes of antimalarial drug resistance genes.

In this study, a *Pf*Ago-triggered dual-signal biosensor combined with recombinase polymerase amplification was developed and evaluated for rapid and sensitive genotyping of the key mutation *Pfcrt*-CVMNK/CVIET haplotype of CQR. The platform can also be applied to identify Plasmodium species and the genotypes of other antimalarial drug resistance genes.

## Methods

### Overall workflow of the RPA-*Pf*Ago platform

**Fig. 1:**
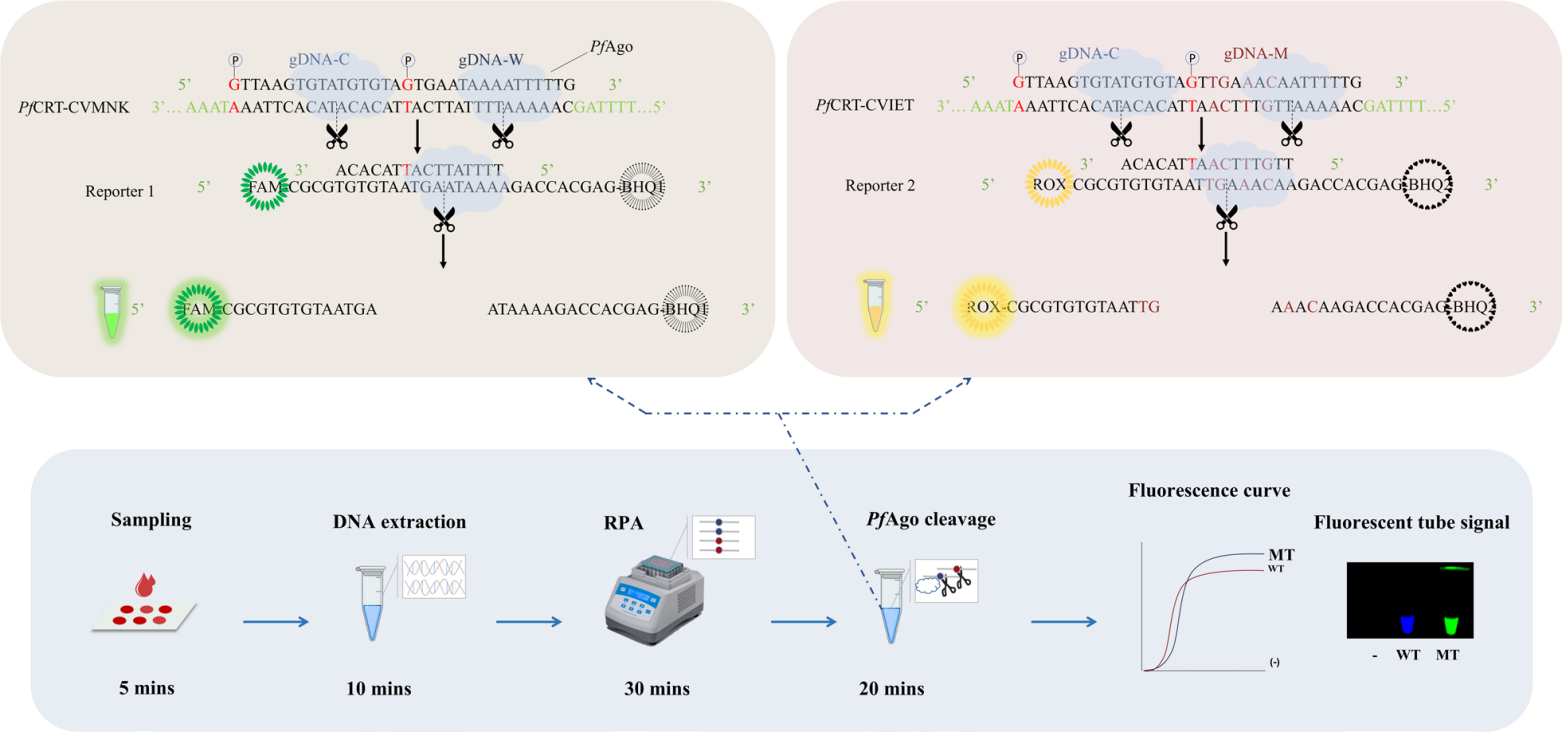
A dual-signal biosensor detection platform triggered by RPA-*Pf*Ago for *Pfcrt* haplotype detection

For rapid and sensitive genotyping of the *Pfcrt* mutant haplotype in the field, a dual-signal biosensor detection platform triggered by RPA combined with *Pf*Ago was developed (Fig. 1). After blood samples were collected from suspected malaria patients, genomic DNA was rapidly extracted via the Chelex-100 method [22]. The target sequence is amplified via RPA technology. The product was subsequently identified by the *Pf*Ago cleavage system. With the guidance of gDNA mutation (gDNA-M) and gDNA-common (gDNA-C), *Pf*Ago can specifically cleave one strand of the amplified product fragment that is complementary to the gDNA-M sequence (the first cleavage). This cleavage produces another ssDNA fragment with 5′ phosphorylation, which serves as the new gDNA to guide *Pf*Ago to perform the second cleavage. The substrate of the second cleavage is the fluorescent probe, the ssDNA reporter; its markers are the ROX fluorophore and the BHQ2 quencher. The fluorescent reporter was designed to confirm the production of the first cleavage. The specific cleavage of the fluorescent reporter by *Pf*Ago releases ROX from BHQ2, producing a fluorescent signal. For the amplified CVMNK fragment, because there is no strand complementary to gDNA-M, the first cleavage cannot occur, and there is no fluorescence signal. Moreover, we designed the corresponding gDNA-wild-type (gDNA-W) strain and another reporter with the FAM fluorophore and the BHQ1 quencher for the wild-type strain. Once the CVMNK amplification product is present, it will produce another kind of fluorescent signal. By using these methods, we detected the CVMNK and CVIET fragments simultaneously in the same reaction mixture containing orthogonal *Pf*Ago proteins, and the orthogonal system yielded two independent channel signals without interfering with each other.

### *Pf*Ago protein expression and purification

The purification of the *Pf*Ago protein (NCBI-Protein ID: WP_011011654.1) was performed following a protocol reported previously [17]. In brief, codon optimization of the *Pf*Ago gene was performed via JCat software (http://www.jcat.de/). The *Pf*Ago gene was subsequently obtained via gene synthesis and subsequently cloned and inserted into the pET28a ( +) plasmid for recombinant protein expression. Finally, the expression of the His-tag fusion protein was induced in the *Escherichia coli* BL21 (DE3) strain with 1 mM IPTG at 37 °C, after which the protein was purified through Ni-affinity chromatography on an AKTA Prime Plus system (GE Healthcare Life Sciences, Boston, MA). Storage buffer (20 mM of Tris–HCl, pH 8.0; 300 mM of NaCl; 0.5 mM of MnCl_2_; 15% (v/v) glycerol) was used to maintain the eluted purified protein, and aliquots were stored at −80 °C for further use.

### Primer design and screening

The sequence of the *Pfcrt* gene (PF3D7_0709000) of the *P*. *falciparum* 3D7 strain was obtained from PlasmoDB (http://plasmodb.org/plasmo/, Release 56, 15 Feb 2022). A series of RPA primers (F1-F3, forward; R1-R3, reverse) were designed according to the RPA design manual (www.twistdx.co.uk) and Primer-BLAST, combining Primer Premier 5.0 software (Premier Biosoft, San Francisco, CA, USA) and BLAST (Basic Local Alignment Search Tool, National Center for Biotechnology Information) global alignment. Three forward primers and three reverse primers were cross-assembled into nine combinations (Table S1) and used for RPA amplification, which was conducted via a TwistAmp^®^ Basic Kit (TwistDx, Cambridge, UK). The 50 μL final reaction mixture contained 2.4 μL of each forward primer and reverse primer (10 μM), 2.0 μL of MgAc (280 mM), 29.5 μL of rehydration buffer, 2.0 μL of genomic DNA, lyophilized enzyme pellets, and free water. The reaction mixture was turned up and down 10 times to ensure full mixing. The reaction time and temperature were 20 min and 37 °C, respectively. Isothermal amplification was carried out on a Life Touch Thermal Cycler (Bioer Technology Co., Ltd., China), after which all amplification products were purified via a TIANquick Midi Purification Kit (TIANGEN Biotech Co., Ltd., Beijing, China) and analyzed on a 2% agarose gel.

### Design and screening of guide DNA and ssDNA reporters

Guide DNA (gDNA) is the key factor in the *Pf*Ago cleavage system because it guides the specific recognition of a particular sequence (in the case of this study, a sequence containing the mutant CVIET fragment) in the target by *Pf*Ago, which in turn activates the nuclease. According to the sequence of the amplification region of the optimal primers, two combinations, 5′-phosphorylated gDNA for *Pf*Ago (16 nt in length) and ssDNA reporters with FAM-BHQ1 or ROX-BHQ2 modifications at the two ends, were manually designed for a total of three groups (Table S1). The ssDNA reporter had a 16 nt region to potentially align to the gDNA cleavage product for the second cleavage. All of the oligonucleotides used are listed in Table S1 and were synthesized by GENEWIZ (Suzhou) Co., Ltd., China. The amplification products of the selected primers were used as target fragments to screen three groups of gDNAs via *Pf*Ago cleavage experiments. The optimal gDNA combination should be able to clearly distinguish between *Pfcrt*-CVMNK and *Pfcrt*-CVIET, and the target fragment should be clearly cleaved.

### Plasmid construction

Two kinds of plasmids containing 515 bp fragments of the *Pfcrt* gene were designed and constructed by GENEWIZ (Suzhou) Co., Ltd., China. The two recombinant plasmids used were *pUC57*/*Pfcrt*-CVMNK (wild-type) and *pUC57*/*Pfcrt*-CVIET (mutant-type). The genes were subsequently validated through Sanger sequencing. DNA concentrations were quantified via a NanoDrop^™^ ND-2000 spectrophotometer (Thermo, Wilmington, USA). The quantity of plasmid DNA was calculated via the following formula: plasmid DNA copy number (copies/μL) = (OD260 × 10^−9^ × 6.02 × 10^23^)/(*n* × 660), where *n* represents the plasmid length. Serial tenfold dilutions were performed with the above plasmids, covering a wide range of concentrations (from 1.8 × 10^4^ to 1.8 × 10^10^ copies/μL).

### RPA*-Pf*Ago platform cleavage assay

By referencing the results of other studies [19, 23–26], the reaction system of the RPA-*Pf*Ago platform was initially established. The 25 μL *Pf*Ago reaction mixture contained 4 μL of purified RPA products, 6 μL (200 U/L) of *Pf*Ago, 2 μL (20 μM) of gDNA-common (gDNA-C1), 1 μL (20 μM) of gDNA-WT (gDNA-W), 1 μL (20 μM) of gDNA-MT (gDNA-M), 1 μL (10 μM) of each signal-producing ssDNA reporter (Probe-W1 and Probe-M1), 3 μL (40 mM) of MnCl_2_, 4 μL of nuclease-free H_2_O and 2 μL of endonuclease reaction buffer (15 mM of Tris/HCl pH 8.0, 250 mM of NaCl). The fully mixed reaction mixture was incubated for 30 min at 95 °C in a SLAN-96S Real-Time PCR Detection System (Shanghai Hongshi Medical Technology Co., Ltd., Shanghai, China), after which the FAM and ROX dual fluorescence signals were recorded at 30 s intervals. After the reaction, the reaction tubes were observed by a BLT GelView 6000Plus (Guangzhou Biolight Biotechnology Co., Ltd., Guangzhou, China) with a blue emission filter (470 nm/520 nm) and a green emission filter (530 nm/600 nm) or directly irradiated by a simple ultraviolet (UV) lamp in dark, confined space and observed with the naked eye.

### Optimization of the *Pf*Ago cleavage reaction

To improve the overall performance of the RPA-*Pf*Ago reaction, the concentrations of gDNA, probes, MnCl2, and *Pf*Ago were optimized (Supplementary Materials). In this study, the optimal conditions were determined by observing the fluorescence curve and fluorescent tube signal. After a series of optimization experiments, as described in the Supporting Information, an integrated RPA-*Pf*Ago cleavage platform was established.

### Urea-denaturing PAGE

The *Pf*Ago cleavage products were verified via a urea-PAGE gel. The urea-PAGE mixture consisted of three layers: the lowest layer was 15% urea-PAGE (2.5 mL of 15% urea-PAGE gel solution, 12.5 μL of 10% APS, 2.5 μL of TEMED); the middle layer was 12% urea-PAGE (12% urea-PAGE gel solution, 4.0 mL; 10% APS, 20.0 μL; TEMED, 4.0 μL); and the top layer was a concentrated gel (urea, 1.47 g; PAA, 350 μL; 5% TBE, 700 μL; APS, 35 μL; and TEMED, 3.5 μL of ddH_2_O added to 3.5 mL). Urea-PAGE was carried out at 200 V for 30 min before use, and the sample was loaded at 200 V for 15 min and 150 V for 30 min. After electrophoresis, the urea-PAGE gel was decolorized in PAGE staining solution (Zhongke Ruitai (Beijing) Biotechnology Co., Ltd., Beijing, China) for 30 min and imaged.

### Nested PCR and sequencing

The classical nested PCR primers targeting *Pfcrt* were synthesized as previously described [27, 28] by GENEWIZ (Suzhou) Co., Ltd., China. TaKaRa Taq^™^ HS Perfect Mix (TaKaRa, Carlsbad, CA) was used as the master mixture, and the mixture was supplemented with each primer at a concentration of 0.2 μM. The 25 μL primary PCR mixture contained 12.5 μL of master mix plus 1 μL of DNA template. 1 L μL of PCR products obtained from the primary round were used as DNA templates for the second round of amplification. The 50 μL reaction mixture included 25 × master mix plus 1 μL of primary PCR product. The reaction conditions for rounds one and two are presented in Table S2. All the PCR products were analyzed via 1.0% agarose gel electrophoresis, and DNA sequencing was performed via an ABI 3730XL automated sequencer (PE Biosystems, CT, USA). Chromas software v2.6.6 was used, and the obtained sequences were aligned via the BioEdit Sequence Alignment Editor (version 7.0.5) for variations.

### Sensitivity validation

The *pUC57/Pfcrt*-CVIET and *pUC57/Pfcrt*-CVMNK plasmids were gradient diluted (concentrations ranging from 1.8 × 10^4^ to 1.8 × 10^10^ copies/μL) and used to validate the sensitivity of the RPA-*Pf*Ago platform. In addition, we mixed the *pUC57/Pfcrt*-CVIET and *pUC57/Pfcrt*-CVMNK plasmids and performed gradient dilutions (concentrations ranging from 1.8 × 10^4^ to 1.8 × 10^10^ copies/μL). The sensitivity of the RPA-*Pf*Ago platform for simultaneous detection of the wild-type and mutant strains in a single reaction system was validated. The RPA-*Pf*Ago platform was validated under optimal conditions. The limit of detection (LOD) was defined as the concentration of the plasmid with the lowest fluorescence signal and the longest reaction time. The sensitivity of the RPA-*Pf*Ago platform was measured in terms of detection thresholds and was also used as an evaluation criterion. In addition, we diluted the concentrations of the *pUC57/Pfcrt*-CVMNK and *pUC57/Pfcrt*-CVIET plasmids to 1.8 × 10^5^ copies/μL, and 1 μL, 3 μL, 5 μL, 7 μL, and 10 μL of the *pUC57/Pfcrt*-CVIET plasmid were mixed with the *pUC57/Pfcrt*-CVMNK plasmid for a total volume of 100 μL. Using these mixed plasmids as templates, the performance of the RPA-*Pf*Ago platform for detecting low-concentration mutant types was evaluated.

### Clinical evaluation

*P*. *falciparum* samples were prepared as described in previous studies [28]. Genomic DNA was extracted via the methods described in the supporting information. Nested PCR followed by Sanger sequencing was used to confirm the PCR genotyping results, and the reaction conditions are presented in Table S2. We subsequently randomly selected 85 samples, including wild-type, mutant, and mixed-type *Pfcrt*, and detected them via the RPA-*Pf*Ago platform. The results were interpreted according to the color of the fluorescent signal and verified by comparison with the sequencing results.

### Data analysis

The experimental data were analyzed with SPSS 22.0 (SPSS, Inc., Chicago, IL, USA). A *P* value < 0.05 indicated a significant difference. The sensitivity was calculated as the number of true positives/ (number of true positives + number of false negatives), and the specificity was calculated as the number of true negatives/ (number of true negatives + number of false positives). The false negative rate was calculated as 1—sensitivity—and the false positive rate was calculated as 1—specificity. The 95% confidence intervals (CI) for sensitivity and specificity were calculated via SPSS 22.0.

## Results

### Confirming the optimal use of the RPA primer and gDNA

**Fig. 2:**
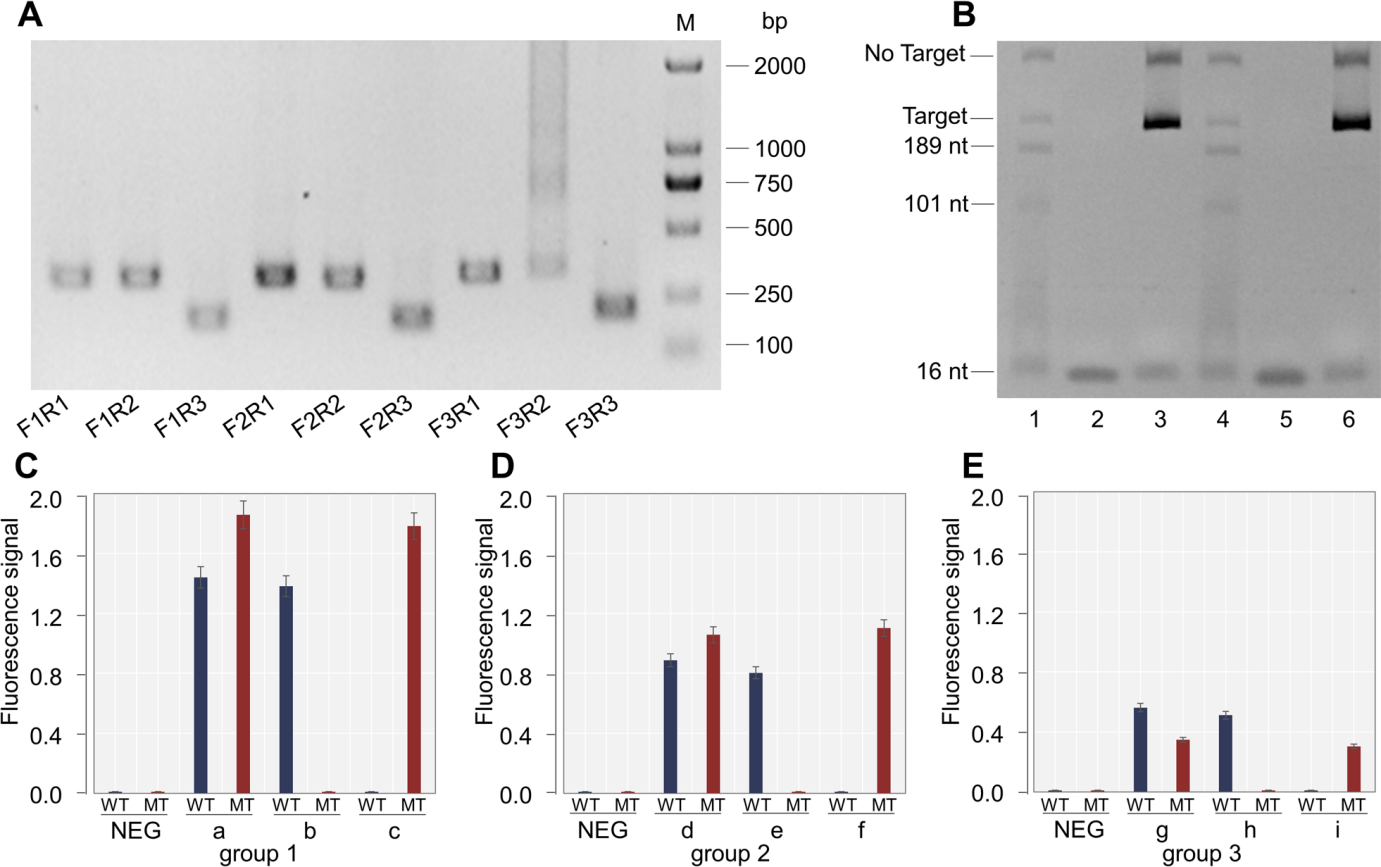
Screening of primers and guide DNA. **A** Screening of RPA primers. **B** Results of urea PAGE of the first group of guide DNAs (gDNAs) after cleavage of the target fragment. Lane 1 is the urea PAGE result of the first gDNA-guided *Pfcrt*-CVMNK cleavage product, lane 2 is the urea PAGE result for gDNA-W only, lane 3 is the urea PAGE result of the *Pfcrt*_CVMNK_-CVMNK amplification products, lane 4 is the urea PAGE result of the first gDNA-guided *Pfcrt*-CVIET cleavage product, lane 5 is the urea PAGE result for gDNA-M only, and lane 6 is the urea PAGE result of the *Pfcrt*-CVIET amplification products. (**C**, **D**, and **E**) The results of the first, second, and third groups of gDNA and reporters, respectively. “a,” “d,” and “g” indicate that the target fragment cleaved by the RPA-PfAgo platform is the amplification product of the wild-type and mutant strains; “b,” “e,” and “h” indicate that the target fragment cleaved by the RPA-PfAgo platform is the amplification product of the wild-type strain; and “c,” “f,” and “i” indicate that the target fragment cleaved by the RPA-PfAgo platform is the amplification product of the mutant strain

The primers (Table S1) used were designed for *Pfcrt* and were cross-assembled into nine combinations. On the gel after RPA, the primer set F2R1 produced single, clear, bright expected bands without nonspecific bands, which is the optimal option (Fig. 2A). Three sets of 5′-phosphorylated gDNAs and ssDNA reporters were subsequently designed and reacted with the RPA amplification products of the F2R1 primer. The results are displayed in Fig. 2C–E. The fluorescence signals of the first group of gDNA were the strongest, and the wild type and the mutant type could be identified. To verify the accuracy of the cleavage, urea PAGE was carried out on the cleavage products of the first set of gDNA. With the guidance of the first set of gDNA, *Pf*Ago can accurately cleave the target product, producing two fragments of different lengths (Fig. 2B).

### Optimization of the *Pf*Ago cleavage reaction

**Fig. 3:**
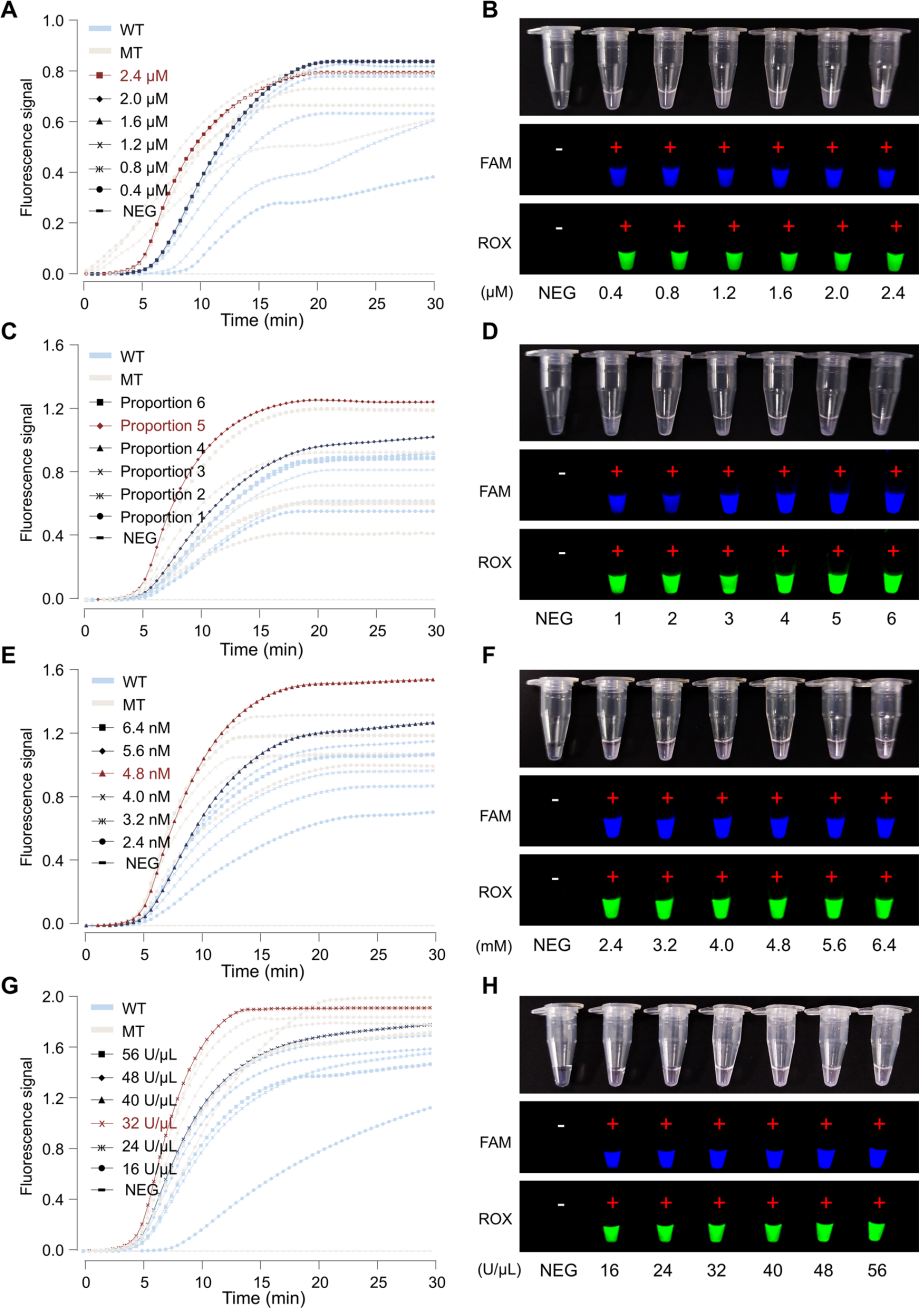
Optimizing *Pf*Ago cleavage conditions. **A** and **B** Optimization of the concentration of guide DNA (gDNA). **C** and **D** Optimizing the concentration proportions of reporter-FAM and reporter-ROX. The numbers “1–6” indicate proportions 1 to 6, respectively. **E** and **F** Optimizing the concentration of MnCl_2_. **G** and **H** Optimizing the addition of *Pf*Ago. **A**, **C**, **E**, and **G** show the fluorescence signal values of the reaction; **B**, **D**, **F**, and **H** show the results of the fluorescent tubes at the end of the reaction

To improve the overall performance of the RPA-*Pf*Ago reaction, the concentrations of gDNA, probes, MnCl_2_, and *Pf*Ago were optimized. Since the fluorescence intensities in the tubes were similar among all the concentrations, the condition with the highest fluorescent signal for the two channel signals at 30 min was selected as the optimal one. gDNA concentrations ranging from 0.4 to 2.4 μM were tested, and fluorescence curves (Fig. 3A) and fluorescent tube signals (Fig. 3B) were observed. The optimal gDNA concentration was 2.4 μM. The reporter concentrations of the reporter-wild-type (Reporter-W) and reporter-mutant (Reporter-M) strains were tested in six groups in different proportions. The fluorescence curve (Fig. 3C) and fluorescence tube signal (Fig. 3D) indicated that 1.2 μM/1.0 μM was the optimal concentration. MnCl_2_ is an activator of the *Pf*Ago cleavage reaction. In this study, six concentrations of MnCl_2_ were used. The fluorescence curve Fig. 3E and fluorescence tube Fig. 3F signals indicated that 4.8 nM was the optimal concentration. Similarly, *Pf*Ago concentrations ranging from 16 to 56 U/μL were tested. Two channel signals achieved a high fluorescence signal simultaneously at 32 U/μL, which was determined to be the optimal concentration Fig. 3G, H.

### Sensitivity of the RPA-*Pf*Ago platform

**Fig. 4:**
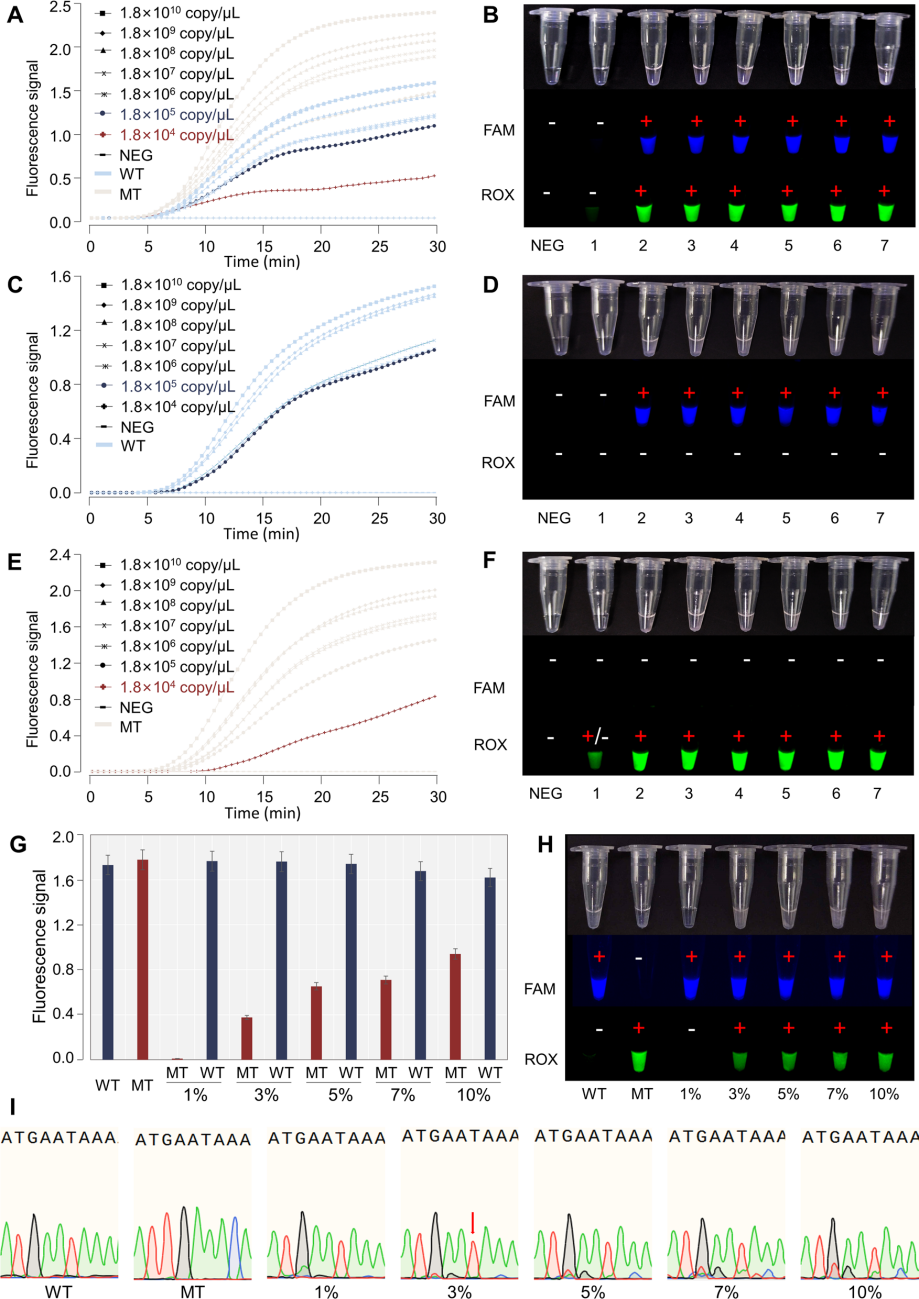
Sensitivity test. **A** and **B** Sensitivity of the *Pfcrt*-CVMNK plasmid. **C** and **D** Sensitivity of the *Pfcrt*-CVIET plasmid. (*E* and *F*) Sensitivity results of the mixed plasmids. The numbers “1–7” indicate 1.8 × 10^4^ to 1.8 × 10^10^ copies/μL, respectively. **G** and **H** Fluorescence curve and fluorescence tube signal for the two-plasmid mixture based on RPA-*Pf*Ago. **I** Sanger sequencing results for the two-plasmid mixture. Red or green bimodal peaks were not observed at the locations indicated by the arrows for 3% of the CVIET mixed plasmids. Extraneous noise occurred in the 5% and 7% CVIET mixed plasmids. **A**, **C**, and **E** show the fluorescence signal values of the reaction; **G** shows the fluorescence signal values at the end of the reaction; **B**, **D**, **F**, and **H** show the results of the fluorescent tubes at the end of the reaction

To explore the sensitivity of the RPA-*Pf*Ago platform, the *pUC57/Pfcrt*-CVIET and *pUC57/Pfcrt*-CVMNK plasmids were tested, and the concentrations ranged from 1.8 × 10^4^ to 1.8 × 10^10^ copies/μL. According to the fluorescence signals, concentrations as low as 1.8 × 10^5^ copies/μL of *pUC57/Pfcrt*-CVMNK (Fig. 4A, B) and 1.8 × 10^4^ copies/μL of *pUC57/Pfcrt*-CVIET (Fig. 4C, D) were detected, which is consistent with the sensitivity of the mixed plasmids (Fig. 4E, F). Furthermore, we diluted the concentrations of the pUC57/*Pfcrt*-CVMNK and *pUC57*/*Pfcrt*-CVIET plasmids to 1.8 × 10^5^ copies/μL, and 1 μL, 3 μL, 5 μL, 7 μL, and 10 μL of the *pUC57/Pfcrt*-CVIET plasmid were mixed with the *pUC57/Pfcrt*-CVMNK plasmid for a total volume of 100 μL. The RPA-*Pf*Ago platform was used, and nPCR was combined with Sanger sequencing to detect the mixed plasmid simultaneously. The RPA-*Pf*Ago platform detected as little as 3% of the *pUC57*/*Pfcrt*-CVIET mixed plasmids (Fig. 4G, H). However, sequencing identified up to 5% of the *pUC57*/*Pfcrt*-CVIET mixed plasmids Fig. 4I.

### Clinical evaluation

**Fig. 5:**
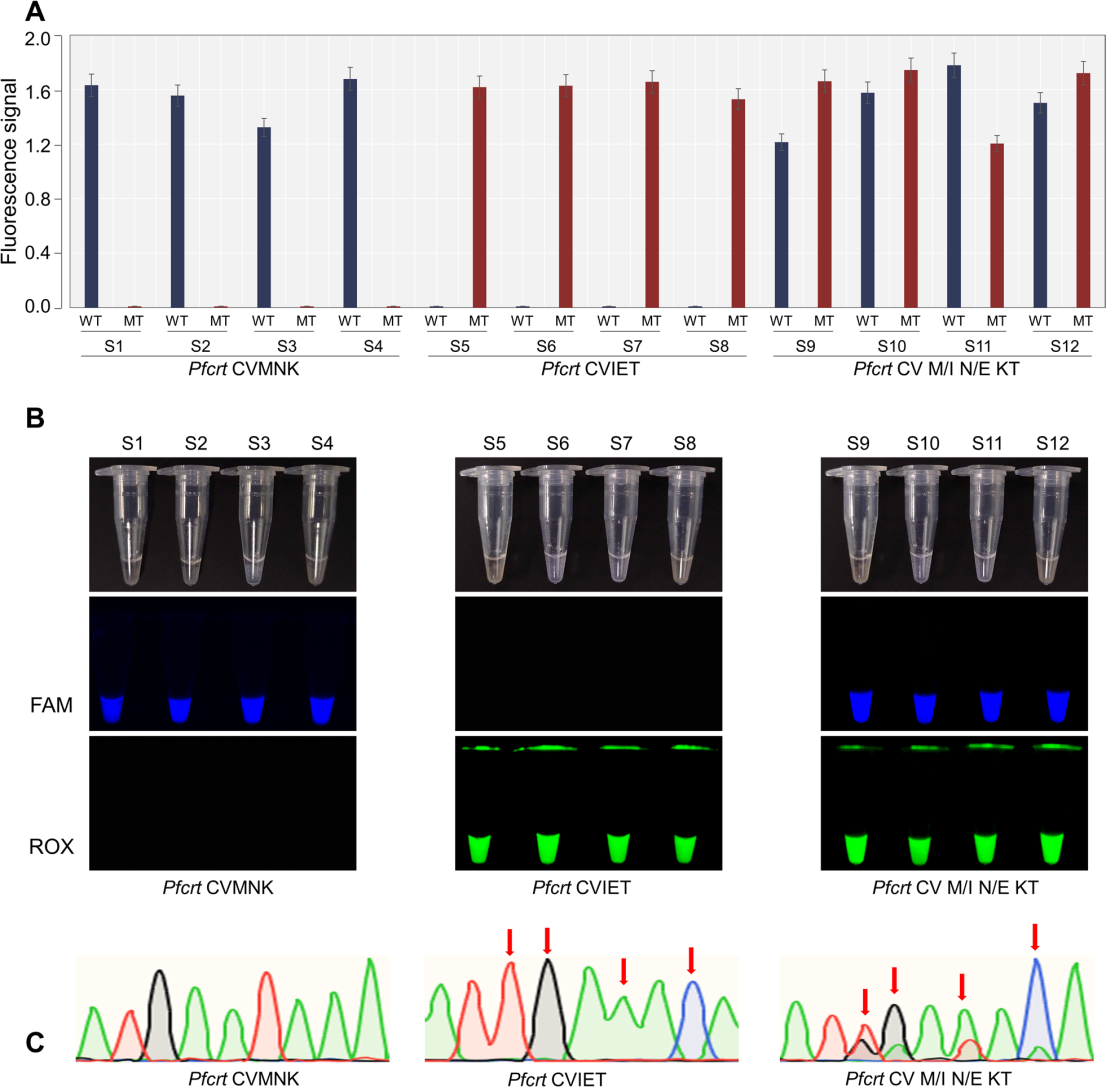
Results for clinical samples (using partial clinical samples as an example). **A** and **B** The results of the *Pfcrt* RPA-*Pf*Ago platform are shown (partially) for three groups of clinical samples: CVMNK, CVIET, and CVM/I N/E K/T. **A** shows the fluorescence signal values at the end of the reaction. **B** shows the results of the fluorescent tubes at the end of the reaction. **C** Typical representation of sequencing results from CVMNK, CVIET, and CVM/I N/E K/T, three different types of clinical samples. The arrows point to the mutated sites

The efficacy of the platform was further confirmed via the use of clinical samples to test its reliability. The ultimate genotyping results for each allele were visually interpreted by changes in the curves on the RPA-*Pf*Ago platform. In total, 85 DBSs were used for clinical evaluation (Table S3). These samples were genotyped via Sanger sequencing; moreover, the RPA-*Pf*Ago platform was used. The results are presented in Fig. 5. When the template was the wild type, reporter-W released a fluorescent signal; when the template was the mutant type, reporter-M released a fluorescent signal; and when the template was the mixed type, two fluorescent signals were generated (partially explaining the samples). The results of the methodological assessment and analysis of the assay results of the RPA-*Pf*Ago platform with the Sanger sequencing results are displayed in Table 1. For these clinical samples, the sensitivity and specificity of CVMNK, CVIET, and the CVM/I N/E K/T were 100%. All the results indicated that the platform was consistent with the nPCR results.

**Table 1:** Methodological comparison of RPA-*Pf*Ago and nested PCR with Sanger sequencing

Haplotype	Method		Sensitivity (%)		Specificity (%)		False negative (%)	False positive (%)
	Nested PCR with Sanger sequencing	RPA-*Pf*Ago	N (%)	95% CI	N (%)	95% CI		
CVMNK	55	55	100	0.9187–1	100	0.8587–1	0.00	0.00
CVIET	19	19	100	0.7908–1	100	0.9315–1	0.00	0.00
CV M/I N/E K/T	11	11	100	0.6786–1	100	0.9385–1	0.00	0.00
Total	85	85	100	0.9461–1	100	NaN	0.00	0.00

## Discussion

For the detection of *Plasmodium* parasite mutations, nPCR combined with sequencing is regarded as the “gold standard” technique [29–31]; this technique is relatively accurate and is used extensively [32, 33]. However, these materials are prone to technological malfunctions [34]. The quality of the PCR products used for sequencing determines the accuracy and reliability of Sanger sequencing results [35], especially concerning the amplification and purification of PCR products before sequencing [34]. However, in clinical samples, when the concentration of the mutation template is low, the quality of the PCR products is not effectively controlled.

Despite the many sequencing standards that have been put forward in recent years to lessen the limitations of Sanger sequencing, several challenges inherent in actual sequencing reads, such as extraneous noise, have not been adequately addressed [35]. An important technical challenge in the interpretation of DNA chromatograms from sequencing data is the existence of extraneous noise in the data. This interference can cause mistakes in base calling and increase the difficulty in distinguishing between real signals and background noise [35]. This was also found in our research (5% and 7% CVIET mixed plasmids, as shown in Fig. 4I). On the other hand, fewer ideal peaks can call for more fine-tuning or troubleshooting to improve the quality of the data [36]. However, fine-tuning may result in the loss of certain low signals. As shown in Fig. 4I, for 3% CVIET mixed plasmids, we did not observe red or green bimodal peaks at the locations indicated by the arrows. Additionally, another technical challenge arises from the double peaks present within DNA chromatograms [35]. These dual peaks might stem from genuine heterozygosity, technical anomalies, or the occurrence of double infections. Determining the characteristics of these double peaks might be difficult, and their existence may cause inaccuracies when performing sequence analysis (5% and 7% CVIET mixed plasmids in Fig. 4I).

Because the RPA-*Pf*Ago platform does not have a unique advantage in the sensitive detection of individual plasmids, it might be unsatisfactory at detecting low-density infections. However, it is satisfactory in clinical applications. The sensitivity and specificity were 100% when 85 clinical samples were used. The methodological advantage of our method is the ability to detect a low proportion of the mutant in mixed infections. The RPA-*Pf*Ago platform achieved 3% sensitivity for genotyping the CVMNK/CVIET mutation of the *Pfcrt* gene, with a higher sensitivity than nPCR combined with Sanger sequencing, which is advantageous over other detection methods (Table 2). An important feature of this platform is its convenience. The amplification step used RPA, which was conducted isothermally at 37 °C; the temperature of *Pf*Ago cleavage was 95 °C [24]. Precise thermal cycling equipment is not needed. The platform only needs minimum laboratory support, such as pipettors, a portable spin, a heat block, a blue-light source or UV light. The entire platform procedure was completed within 1.5 h. Because the *Pf*Ago enzyme is thermostable and the RPA reagent is lyophilized, the reagents used in this platform do not require cold chain transport or storage. The RPA-*Pf*Ago platform is a rapid and sensitive genotyping platform for *Pfcrt* that can be easily applied locally or on site [14].

**Table 2:** Molecular methods used for identifying mutations in antimalarial drug resistance genes

Classification	Method	LOD	Thermocycler	Advantages	Disadvantages	References
PCR-based assays	mPCR	20 fg/μL	Yes	High accuracy, high sensitivity, time savings, and economic advantages	The quality of the probe is significantly influenced by the base sequence near the detection site	[1]
	HRM	0.3 ng/μl	Yes	Simple, robust, low-cost	Amplicons with sizes greater than 300 bps may produce more errors	[2–4]
	AS-PCR	50 parasites/μL	Yes	Most common; easiest	Nonspecific amplification often occurs	[5, 6]
	RFLP	15 parasites/μl	Yes	Rapid, simple, convenient, and inexpensive	Not suitable for large-scale testing	[7–9]
Isothermal tests	RPA	3.17 ng/μl	No	No thermocycler required rapid	Susceptible to laboratory contamination; sensitivity not as good as qPCR	[10, 11]
	LFA	3.38 × 10^5^ copies/μL	No	Easy to interpret test results	Susceptible to laboratory contamination	[5, 6, 11]
	RPA-*Pf*Ago	3%	No	No thermocycler needed; rapid; simple to understand the results of the test	The RPA amplification products need to be purified by the purification kit to remove the proteins involved in the RPA reaction	
Sequencing methods	Sanger sequencing	20%	Yes	Provide thorough haplotype information	Time-consuming and extensive data analysis requires costly equipment and well-trained technicians	[12]
	Illumina MiSeq	5%	Yes	Provide thorough haplotype information; faster than Sanger sequencing	Requires expensive equipment and well-trained technicians; and extensive data analysis	[13]

LOD means limit of detection

The RPA-*Pf*Ago platform not only has wide application value in resource-poor areas and clinical research but also offers a feasible platform for haplotype detection [14, 23]. The other advantage of the RPA-*Pf*Ago platform is that it can detect CVMNK and the mutant CVIET simultaneously via a dual-signal biosensor system. Monitoring population changes and controlling malaria epidemics should benefit greatly from the identification of mixed infections via an integrated test.

However, the RPA amplification products must be purified by a purification kit (centrifugal column type) to remove the proteins, ions, and other impurities involved in the RPA reaction, which requires slightly tedious steps. Otherwise, *Pf*Ago cleavage will not occur, which reduces the portability of the POCT method. We have tested existing rapid purification methods; however, a rapid purification method adapted to our platform has yet to be found. For example, organic solvent residues in the RPA product are purified via the phenol‒chloroform extraction method, which denatures the *Pf*Ago enzyme and prevents cleavage from occurring. The thermal denaturation method can only denature and inactivate the recombinase and polymerase by heat but fails to achieve complete separation of the protein, ions, and other impurities from the DNA amplification product by high-speed centrifugation, which affects the *Pf*Ago reaction system and prevents the digestion reaction from occurring [37]. We will focus on solving this problem and making the POCT method more portable in future research.

## Conclusions

This study established an RPA-*Pf*Ago platform for genotyping the key mutation *Pfcrt*-CVMNK/CVIET of CQR. This approach has the characteristics of a short operation time, low device dependence, and good match to the POCT strategy, suggesting that the platform can be easily applied locally or on site. These findings strongly support the epidemiological investigations of *P*. *falciparum*, as they can not only be used to indicate resistance to CQ and other 4-aminoquinoline antimalarial drugs but also be used to monitor population changes in *P*. *falciparum*.

## Supplementary Information

Supplementary Material 1.

## Data Availability

All the data generated or analyzed during this study are included in this published article and its supplementary information files.

## Abbreviations

*Pf*Ago: *Pyrococcus furiosus* Argonaute
*Pf*crt: *Plasmodium falciparum* chloroquine resistance transporter
POCT: Point-of-care testing
RPA: Recombinase polymerase amplification
SNP: Single-nucleotide polymorphism
CQ: Chloroquine
CQR: Chloroquine resistance
nPCR: Nest-PCR
PCR–RFLP: PCR-restriction fragment length polymorphism
AS‒PCR: Allele-specific diagnostic PCR
PAM: Protospacer-adjacent motif
PFS: Protospacer flanking site

## References

[1] WHO. World malaria report. Geneva: World Health Organization; 2020.

[2] Cheng W, Song X, Zhu H, Wu K, Wang W, Li J. A rapid and specific genotyping platform for *Plasmodium falciparum* chloroquine resistance via allele-specific pcr with a lateral flow assay. Microbiol Spectr. 2022;10:e0271921. 35416696 10.1128/spectrum.02719-21 PMC9045167

[3] Sowunmi A, Ayede AI, Falade AG, Ndikum VN, Sowunmi CO, Adedeji AA, et al. Randomized comparison of chloroquine and amodiaquine in the treatment of acute, uncomplicated, *Plasmodium* falciparum malaria in children. Ann Trop Med Parasitol. 2001;95:549–58. 11672461 10.1080/00034980120092507

[4] Alifrangis M, Dalgaard MB, Lusingu JP, Vestergaard LS, Staalsoe T, Jensen AT, et al. Occurrence of the southeast Asian/South American SVMNT haplotype of the chloroquine-resistance transporter gene in *Plasmodium falciparum* in Tanzania. J Infect Dis. 2006;193:1738–41. 16703518 10.1086/504269

[5] Patel P, Bharti PK, Bansal D, Ali NA, Raman RK, Mohapatra PK, et al. Prevalence of mutations linked to antimalarial resistance in *Plasmodium falciparum* from Chhattisgarh, central India: a malaria elimination point of view. Sci Rep. 2017;7:16690. 29192183 10.1038/s41598-017-16866-5 PMC5709362

[6] Xu C, Wei Q, Yin K, Sun H, Li J, Xiao T, et al. Surveillance of antimalarial resistance Pfcrt, Pfmdr1, and Pfkelch13 polymorphisms in African *Plasmodium falciparum* imported to Shandong Province, China. Sci Rep. 2018;8:12951. 30154519 10.1038/s41598-018-31207-w PMC6113250

[7] Rei Yan SL, Wakasuqui F, Wrenger C. Point-of-care tests for malaria: speeding up the diagnostics at the bedside and challenges in malaria cases detection. Diagn Microbiol Infect Dis. 2020;98:115122. 32711185 10.1016/j.diagmicrobio.2020.115122

[8] Cheng W, Wang W, Zhu H, Song X, Wu K, Li J. Detection of antimalarial resistance-associated mutations in *Plasmodium falciparum* via a platform of allele-specific PCR combined with a gold nanoparticle-based lateral flow assay. Microbiol Spectr. 2022;10:e0253522. 36445076 10.1128/spectrum.02535-22 PMC9769821

[9] Hänscheid T, Grobusch MP. How useful is PCR in the diagnosis of malaria? Trend Parasitol. 2002;18:395–8. 10.1016/s1471-4922(02)02348-6 12377256

[10] Mens PF, van Amerongen A, Sawa P, Kager PA, Schallig HD. Molecular diagnosis of malaria in the field: development of a novel 1-step nucleic acid lateral flow immunoassay for the detection of all 4 human *Plasmodium* spp. and its evaluation in Mbita. Kenya Diagn Microbiol Infect Dis. 2008;61:421–7. 18455349 10.1016/j.diagmicrobio.2008.03.009

[11] Chahar M, Mishra N, Anvikar A, Dixit R, Valecha N. Establishment and application of a novel isothermal amplification assay for rapid detection of chloroquine resistance (K76T) in *Plasmodium falciparum*. Sci Rep. 2017;7:41119. 28134241 10.1038/srep41119 PMC5278370

[12] Huang M, Zhou X, Wang H, Xing D. Clustered regularly interspaced short palindromic repeats/Cas9 triggered isothermal amplification for site-specific nucleic acid detection. Anal Chem. 2018;90:2193–200. 29260561 10.1021/acs.analchem.7b04542

[13] Chahar M, Anvikar A, Dixit R, Valecha N. Evaluation of four novel isothermal amplification assays towards simple and rapid genotyping of chloroquine resistant *Plasmodium falciparum*. Exp Parasitol. 2018;190:1–9. 29750967 10.1016/j.exppara.2018.05.001

[14] Zhao C, Yang L, Zhang X, Tang Y, Wang Y, Shao X, et al. Rapid and sensitive genotyping of SARS-CoV-2 Key mutation L452R with an RPA-PfAgo method. Anal Chem. 2022;94:17151–9. 36459151 10.1021/acs.analchem.2c03563

[15] Olovnikov I, Chan K, Sachidanandam R, Newman DK, Aravin AA. Bacterial argonaute samples the transcriptome to identify foreign DNA. Mol Cell. 2013;51:594–605. 24034694 10.1016/j.molcel.2013.08.014 PMC3809076

[16] Swarts DC, Jore MM, Westra ER, Zhu Y, Janssen JH, Snijders AP, et al. DNA-guided DNA interference by a prokaryotic Argonaute. Nature. 2014;507:258–61. 24531762 10.1038/nature12971 PMC4697943

[17] Swarts DC, Hegge JW, Hinojo I, Shiimori M, Ellis MA, Dumrongkulraksa J, et al. Argonaute of the archaeon *Pyrococcus furiosus* is a DNA-guided nuclease that targets cognate DNA. Nucl Acid Res. 2015;43:5120–9. 10.1093/nar/gkv415 PMC4446448 25925567

[18] Enghiad B, Zhao H. Programmable DNA-guided artificial restriction enzymes. ACS Synth Biol. 2017;6:752–7. 28165224 10.1021/acssynbio.6b00324

[19] Ye X, Zhou H, Guo X, Liu D, Li Z, Sun J, et al. Argonaute-integrated isothermal amplification for rapid, portable, multiplex detection of SARS-CoV-2 and influenza viruses. Biosens Bioelectron. 2022;207:114169. 35334329 10.1016/j.bios.2022.114169 PMC9759211

[20] Cao Y, Yisimayi A, Jian F, Song W, Xiao T, Wang L, et al. BA2121, BA4 and BA5 escape antibodies elicited by omicron infection. Nature. 2022;608:593–602. 35714668 10.1038/s41586-022-04980-y PMC9385493

[21] Nagasawa N, Kimura R, Akagawa M, Shirai T, Sada M, Okayama K, et al. Molecular evolutionary analyses of the spike protein gene and spike protein in the SARS-CoV-2 omicron subvariants. Microorganisms. 2023;11(9):2336. 10.3390/microorganisms11092336. 37764181 10.3390/microorganisms11092336 PMC10537508

[22] Li J, Chen JT, Xie DD, Monte-Nguba SM, Eyi JUM, Matesa RA, et al. High prevalence of pfmdr1 N86Y and Y184F mutations in *Plasmodium falciparum* isolates from Bioko Island, Equatorial Guinea. Pathog Glob Health. 2014;108:339–43. 25348116 10.1179/2047773214Y.0000000158 PMC4241786

[23] Wang F, Yang J, He R, Yu X, Chen S, Liu Y, et al. PfAgo-based detection of SARS-CoV-2. Biosens Bioelectron. 2021;177:112932. 33429204 10.1016/j.bios.2020.112932 PMC7832551

[24] Liu Q, Guo X, Xun G, Li Z, Chong Y, Yang L, et al. Argonaute integrated single-tube PCR system enables supersensitive detection of rare mutations. Nucleic Acids Res. 2021;49:e75. 33905513 10.1093/nar/gkab274 PMC8287959

[25] Yang L, Guo B, Wang Y, Zhao C, Zhang X, Wang Y, et al. *Pyrococcus furiosus* Argonaute combined with recombinase polymerase amplification for rapid and sensitive detection of Enterocytozoon hepatopenaei. J Agric Food Chem. 2023;71:944–51. 36548210 10.1021/acs.jafc.2c06582

[26] Li Y, Kou J, Han X, Qiao J, Zhang W, Man S, et al. Argonaute-triggered visual and rebuilding-free foodborne pathogenic bacteria detection. J Hazard Mater. 2023;454:131485. 37149945 10.1016/j.jhazmat.2023.131485

[27] Li J, Chen J, Xie D, Eyi UM, Matesa RA, Obono MMO, et al. Molecular mutation profile of Pfcrt and Pfmdr1 in *Plasmodium falciparum* isolates from Bioko Island, Equatorial Guinea. Infection Genet Evolut. 2015;36:552–6. 10.1016/j.meegid.2015.08.039 26325683

[28] Liu Y, Liang X, Li J, Chen J, Huang H, Zheng Y, et al. Molecular surveillance of artemisinin-based combination therapies resistance in *Plasmodium falciparum* parasites from Bioko Island, Equatorial Guinea. Microbiol Spect. 2022;10:e0041322. 10.1128/spectrum.00413-22 PMC9241599 35670601

[29] Yao Y, Wu K, Xu M, Yang Y, Zhang Y, Yang W, et al. Surveillance of genetic variations associated with antimalarial resistance of *Plasmodium falciparum* isolates from returned migrant workers in Wuhan, Central China. Antimicrob Agent Chemother. 2018;62(9):e02387–17. 10.1128/AAC.02387-17. 10.1128/AAC.02387-17 PMC6125563 29941645

[30] Feng J, Xu D, Kong X, Lin K, Yan H, Feng X, et al. Characterization of pfmdr1, pfcrt, pfK13, pfubp1, and pfap2mu in travelers returning from Africa with *Plasmodium falciparum* infections reported in China from 2014 to 2018. Antimicrob Agent Chemother. 2021;65:e0271720. 10.1128/AAC.02717-20 PMC8218672 33903109

[31] Cheng W, Song X, Tan H, Wu K, Li J. Molecular surveillance of anti-malarial resistance pfcrt, pfmdr1, and pfk13 polymorphisms in African *Plasmodium falciparum* imported parasites to Wuhan, China. Malar J. 2021;20:209. 33933099 10.1186/s12936-021-03737-8 PMC8087876

[32] Beck TF, Mullikin JC, Biesecker LG. Systematic evaluation of sanger validation of next-generation sequencing variants. Clin Chem. 2016;62:647–54. 26847218 10.1373/clinchem.2015.249623 PMC4878677

[33] Lawi ZK, Al-Shuhaib MBS, Amara IB, Alkhammas AH. Two missense variants of the epidermal growth factor receptor gene are associated with non small cell lung carcinoma in the subjects from Iraq. Mol Biol Rep. 2022;49:11653–61. 36169894 10.1007/s11033-022-07933-w

[34] Schirmer M, D’Amore R, Ijaz UZ, Hall N, Quince C. Illumina error profiles: resolving fine-scale variation in metagenomic sequencing data. BMC Bioinform. 2016;17:125. 10.1186/s12859-016-0976-y PMC4787001 26968756

[35] Al-Shuhaib MBS, Hashim HO. Mastering DNA chromatogram analysis in Sanger sequencing for reliable clinical analysis. J Genet Eng Biotechnol. 2023;21:115. 37955813 10.1186/s43141-023-00587-6 PMC10643650

[36] Slatko BE, Gardner AF, Ausubel FM. Overview of next-generation sequencing technologies. Curr Protoc Mol Biol. 2018;122:e59. 29851291 10.1002/cpmb.59 PMC6020069

[37] Londoño MA, Harmon CL, Polston JE. Evaluation of recombinase polymerase amplification for detection of begomoviruses by plant diagnostic clinics. VIROL J. 2016;13:48. 27000806 10.1186/s12985-016-0504-8 PMC4802622

