# Supplementary material for: Rapid and supersensitive allele detection of Plasmodium falciparum chloroquine resistance via a Pyrococcus furiosus argonaute-triggered dual-signal biosensing platform
Source: Parasit Vectors. 2024 Nov 24;17:488. doi: 10.1186/s13071-024-06575-0 (PMC11587582; doi:10.1186/s13071-024-06575-0)
Supplement: Supplementary file 1 — Supplementary Material 1. [file 13071_2024_6575_MOESM1_ESM.doc]

**Supplementary information**

# 1. Materials

1.1 DNA extraction

Genomic DNA was extracted from dried blood spots (DBSs) with a Genomic DNA Extraction Kit for Dry Blood Spot (No. DP334, TIANGENE Biotech [Beijing] Co., Ltd.), following the manufacturer’s protocol. The extracted gDNA was quantified with a NanoDrop^TM^ ND-2000 spectrophotometer (Thermo, Wilmington, USA) and then stored at -80°C until further analysis.

1.2 Optimizing the *Pf*Ago cleavage reaction

To improve the overall performance of the RPA-*Pf*Ago reaction, the concentrations of gDNA, probes, MnCl2, and *Pf*Ago were optimized under the reaction conditions. The best primers were used for RPA amplification, and the optimal reaction conditions were determined by the PfAgo cleavage reaction. First, six different gDNA concentrations, ranging from 0.4 to 2.4 μM, were tested: 0.4 μM, 0.8 μM, 1.2 μM, 1.6 μM, 2.40 μM, and 2.4 μM. The optimal concentration was determined by observing the fluorescence curve and fluorescent tube signal. Second, the reporter concentrations of the reporter-wild-type (Reporter-W) and reporter-mutated (Reporter-M) strains were tested in six groups with different proportions. The proportions of bacteria at concentrations of 1 to 6 were 0.4 μM/0.2 μM, 0.4 μM/0.4 μM, 0.8 μM/0.4 μM, 1.2 μM/0.5 μM, 1.2 μM/1.0 μM and 1.6 μM/0.8 μM, respectively. Third, MnCl_2_ is an activator of the *Pf*Ago cleavage reaction. In this study, six concentrations of MnCl2 were tested: 2.4 nM, 3.2 nM, 4.0 nM, 4.8 nM, 5.6 nM, and 6.4 nM. Finally, six different *Pf*Ago concentrations ranging from 16 to 56 U/μL were tested: 16 U/μL, 24 U/μL, 32 U/μL, 40 U/μL, 48 U/μL, and 56 U/μL.

1.3 Plasmid sequences

*pUC57/Pfcrt*_CVIET_: ATATATATATATATATATGTATACCCATATGTATTAATTTTTTTTTTTTTTTTTTTTTTTTTTTTTTTTTTTTTTCCCTTGTCGACCTTAACAGATGGCTCACGTTTAGGTGGAGGTTCTTGTCTTGGTAAATGTGCTCATGTGTTTAAACTTATTTTTAAAGAGATTAAGGATAATATTTTTATTTATATTTTAAGTATTATTTATTTAAGTGTATGTGTAATTGAAACAATTTTTGCTAAAAGAACTTTAAACAAAATTGGTAACTATAGTTTTGTAACATCCGAAACTCACAACTTTATTTGTATGATTATGTTCTTTATTGTTTATTCCTTATTTGGAAATAAAAAGGGAAATTCAAAAGTAAGATAAATCAATATATTAAAATGATGGATTTATAAGAGAATCTATTCCACCTACCAATATAAAACATTACACATATATATATATATATATATATATATATATATATATATATATATATGTATGTATGTTGATTAATTTGTTTATATATT

*pUC57/Pfcrt*_CVMNK_: ATATATATATATATATATGTATACCCATATGTATTAATTTTTTTTTTTTTTTTTTTTTTTTTTTTTTTTTTTTTTCCCTTGTCGACCTTAACAGATGGCTCACGTTTAGGTGGAGGTTCTTGTCTTGGTAAATGTGCTCATGTGTTTAAACTTATTTTTAAAGAGATTAAGGATAATATTTTTATTTATATTTTAAGTATTATTTATTTAAGTGTATGTGTAATGAATAAAATTTTTGCTAAAAGAACTTTAAACAAAATTGGTAACTATAGTTTTGTAACATCCGAAACTCACAACTTTATTTGTATGATTATGTTCTTTATTGTTTATTCCTTATTTGGAAATAAAAAGGGAAATTCAAAAGTAAGATAAATCAATATATTAAAATGATGGATTTATAAGAGAATCTATTCCACCTACCAATATAAAACATTACACATATATATATATATATATATATATATATATATATATATATATATATGTATGTATGTTGATTAATTTGTTTATATATT

# 2. Supplementary Figures and Tables

Table S1 Sequences of the primers, probes and gDNA

Table S2 The conditions for nested PCR of *pfcrt* gene.

Table S3 Comparison between sequencing and RPA-PfAgo for SNP detection in the *pfcrt* gene with clinical isolates.

Table S1 Sequences of the primers, probes and gDNA

| Type | Group | Name | Sequence (5'-3') |
| --- | --- | --- | --- |
| RPA |  | F1 | GTGGAGGTTCTTGTCTTGGTAAATGTGCTC |
|  |  | F2 | GTTCTTGTCTTGGTAAATGTGCTCATGTG |
|  |  | F3 | CAGATGGCTCACGTTTAGGTGGAGGTTCTTGTC |
|  |  | R1 | GGTAGGTGGAATAGATTCTCTTATAAATCC |
|  |  | R2 | GGTAGGTGGAATAGATTCTCTTATAAATCCATC |
|  |  | R3 | GTTGTGAGTTTCGGATGTTACAAAACTATAG |
| Nested PCR | round one | *pfcrt*-F1 | CCGTTAATAATAAATACACGCAG |
|  |  | *pfcrt*-R1 | CGGATGTTACAAAACTATAGTTACC |
|  | round two | *pfcrt*-F2 | TGTGCTCATGTGTTTAAACTT |
|  |  | *pfcrt*-R2 | CAAAACTATAGTTACCAATTTTG |
| *Pf*Ago | group 1 | gDNA1-W1 | P-GTGAATAAAATTTTTG |
|  |  | gDNA1-M1 | P-GTTGAAACAATTTTTG |
|  |  | gDNA2-C1 | P-GTTAAGTGTATGTGTA |
|  |  | Reporter-W1 | FAM-CGCGTGTGTAATGAATAAAAGACCACGAG-BHO1 |
|  |  | Reporter-M1 | ROX-CGCGTGTGTAATTGAAACAAGACCACAGC-BHQ2 |
|  | group 2 | gDNA1-W2 | P-GTTTATTCATTACACA |
|  |  | gDNA1-M2 | P-GTGTTTCAATTACACA |
|  |  | gDNA2-C2 | P-ATTCTTTTAGCAAAAA |
|  |  | Reporter-W2 | FAM-GCGTCAAAAATTTTATTCATCCGCGACGT-BHQ1 |
|  |  | Reporter-M2 | ROX-GCGTCAAAAATTGTTTCAATCCGCGACGT-BHQ2 |
|  | group 3 | gDNA1-W3 | P-GGTAATGAATAAAATT |
|  |  | gDNA1-M3 | P-GGTAATTGAAACAATT |
|  |  | gDNA2-C3 | P-GTTATTTAAGTGTATG |
|  |  | Reporter-W3 | FAM-CGCGTGTATGTGTAATGAATGACCACGAG-BHQ1 |
|  |  | Reporter-M3 | ROX-CGCGTGTATGTGTAATTGAAGTCTACGCG-BHQ2 |

Table S2 The conditions for nested PCR of *pfcrt* gene.

| **nested PCR** | **Temperature (°C)** | **Time** | **Cycle** |
| --- | --- | --- | --- |
| Primary round | 95 | 2 min | 1 |
|  | 95 | 30 sec | 30 |
|  | 53 | 30 sec |  |
|  | 72 | 1 min |  |
|  | 72 | 2 min | 1 |
| Second round | 95 | 2 min | 1 |
|  | 95 | 30 sec | 35 |
|  | 53 | 30 sec |  |
|  | 72 | 1 min |  |
|  | 72 | 2 min | 1 |

Table S3 Comparison between sequencing and RPA-PfAgo for SNP detection in the *pfcrt* gene with clinical isolates.

| **Sample No.** | **Nested PCR with sequencing** | | | | | | | | | | |  |  | **RPA-*Pf*Ago** | | | | | | | | | | | |
| --- | --- | --- | --- | --- | --- | --- | --- | --- | --- | --- | --- | --- | --- | --- | --- | --- | --- | --- | --- | --- | --- | --- | --- | --- | --- |
|  | **M74I** | | |  | **N75E** | | |  | **K76T** | | | **Haplotype** |  | **M74I** | | |  | **N75E** | | |  | **K76T** | | | **Haplotype** |
|  | **A220** | **T221** | **G222T** |  | **A223G** | **A224** | **T225A** |  | **A226** | **A227C** | **A228** |  |  | **A220** | **T221** | **G222T** |  | **A223G** | **A224** | **T225A** |  | **A226** | **A227C** | **A228** |  |
| 1 | A | T | G |  | A | A | T |  | A | A | A | CVMNK |  | A | T | G |  | A | A | T |  | A | A | A | CVMNK |
| 2 | A | T | G |  | A | A | T |  | A | A | A | CVMNK |  | A | T | G |  | A | A | T |  | A | A | A | CVMNK |
| 3 | A | T | G |  | A | A | T |  | A | A | A | CVMNK |  | A | T | G |  | A | A | T |  | A | A | A | CVMNK |
| 4 | A | T | G |  | A | A | T |  | A | A | A | CVMNK |  | A | T | G |  | A | A | T |  | A | A | A | CVMNK |
| 5 | A | T | T |  | G | A | A |  | A | C | A | CVIET |  | A | T | T |  | G | A | A |  | A | C | A | CVIET |
| 6 | A | T | T |  | G | A | A |  | A | C | A | CVIET |  | A | T | T |  | G | A | A |  | A | C | A | CVIET |
| 7 | A | T | T |  | G | A | A |  | A | C | A | CVIET |  | A | T | T |  | G | A | A |  | A | C | A | CVIET |
| 8 | A | T | T |  | G | A | A |  | A | C | A | CVIET |  | A | T | T |  | G | A | A |  | A | C | A | CVIET |
| 9 | A | T | G/T |  | A/G | A | T/A |  | A | A/C | A | CV M/I N/E K/T |  | A | T | G/T |  | A/G | A | T/A |  | A | A/C | A | CV M/I N/E K/T |
| 10 | A | T | G/T |  | A/G | A | T/A |  | A | A/C | A | CV M/I N/E K/T |  | A | T | G/T |  | A/G | A | T/A |  | A | A/C | A | CV M/I N/E K/T |
| 11 | A | T | G/T |  | A/G | A | T/A |  | A | A/C | A | CV M/I N/E K/T |  | A | T | G/T |  | A/G | A | T/A |  | A | A/C | A | CV M/I N/E K/T |
| 12 | A | T | G/T |  | A/G | A | T/A |  | A | A/C | A | CV M/I N/E K/T |  | A | T | G/T |  | A/G | A | T/A |  | A | A/C | A | CV M/I N/E K/T |
| 13 | A | T | G/T |  | A/G | A | T/A |  | A | A/C | A | CV M/I N/E K/T |  | A | T | G/T |  | A/G | A | T/A |  | A | A/C | A | CV M/I N/E K/T |
| 14 | A | T | T |  | G | A | A |  | A | C | A | CVIET |  | A | T | T |  | G | A | A |  | A | C | A | CVIET |
| 15 | A | T | G/T |  | A/G | A | T/A |  | A | A/C | A | CV M/I N/E K/T |  | A | T | G/T |  | A/G | A | T/A |  | A | A/C | A | CV M/I N/E K/T |
| 16 | A | T | T |  | G | A | A |  | A | C | A | CVIET |  | A | T | T |  | G | A | A |  | A | C | A | CVIET |
| 17 | A | T | G/T |  | A/G | A | T/A |  | A | A/C | A | CV M/I N/E K/T |  | A | T | G/T |  | A/G | A | T/A |  | A | A/C | A | CV M/I N/E K/T |
| 18 | A | T | G/T |  | A/G | A | T/A |  | A | A/C | A | CV M/I N/E K/T |  | A | T | G/T |  | A/G | A | T/A |  | A | A/C | A | CV M/I N/E K/T |
| 19 | A | T | T |  | G | A | A |  | A | C | A | CVIET |  | A | T | T |  | G | A | A |  | A | C | A | CVIET |
| 20 | A | T | G |  | A | A | T |  | A | A | A | CVMNK |  | A | T | G |  | A | A | T |  | A | A | A | CVMNK |
| 21 | A | T | T |  | G | A | A |  | A | C | A | CVIET |  | A | T | T |  | G | A | A |  | A | C | A | CVIET |
| 22 | A | T | G/T |  | A/G | A | T/A |  | A | A/C | A | CV M/I N/E K/T |  | A | T | G/T |  | A/G | A | T/A |  | A | A/C | A | CV M/I N/E K/T |
| 23 | A | T | T |  | G | A | A |  | A | C | A | CVIET |  | A | T | T |  | G | A | A |  | A | C | A | CVIET |
| 24 | A | T | G |  | A | A | T |  | A | A | A | CVMNK |  | A | T | G |  | A | A | T |  | A | A | A | CVMNK |
| 25 | A | T | G |  | A | A | T |  | A | A | A | CVMNK |  | A | T | G |  | A | A | T |  | A | A | A | CVMNK |
| 26 | A | T | T |  | G | A | A |  | A | C | A | CVIET |  | A | T | T |  | G | A | A |  | A | C | A | CVIET |
| 27 | A | T | T |  | G | A | A |  | A | C | A | CVIET |  | A | T | T |  | G | A | A |  | A | C | A | CVIET |
| 28 | A | T | G |  | A | A | T |  | A | A | A | CVMNK |  | A | T | G |  | A | A | T |  | A | A | A | CVMNK |
| 29 | A | T | G |  | A | A | T |  | A | A | A | CVMNK |  | A | T | G |  | A | A | T |  | A | A | A | CVMNK |
| 30 | A | T | G |  | A | A | T |  | A | A | A | CVMNK |  | A | T | G |  | A | A | T |  | A | A | A | CVMNK |
| 31 | A | T | G |  | A | A | T |  | A | A | A | CVMNK |  | A | T | G |  | A | A | T |  | A | A | A | CVMNK |
| 32 | A | T | T |  | G | A | A |  | A | C | A | CVIET |  | A | T | T |  | G | A | A |  | A | C | A | CVIET |
| 33 | A | T | G |  | A | A | T |  | A | A | A | CVMNK |  | A | T | G |  | A | A | T |  | A | A | A | CVMNK |
| 34 | A | T | G |  | A | A | T |  | A | A | A | CVMNK |  | A | T | G |  | A | A | T |  | A | A | A | CVMNK |
| 35 | A | T | G |  | A | A | T |  | A | A | A | CVMNK |  | A | T | G |  | A | A | T |  | A | A | A | CVMNK |
| 36 | A | T | G/T |  | A/G | A | T/A |  | A | A/C | A | CV M/I N/E K/T |  | A | T | G/T |  | A/G | A | T/A |  | A | A/C | A | CV M/I N/E K/T |
| 37 | A | T | G |  | A | A | T |  | A | A | A | CVMNK |  | A | T | G |  | A | A | T |  | A | A | A | CVMNK |
| 38 | A | T | G |  | A | A | T |  | A | A | A | CVMNK |  | A | T | G |  | A | A | T |  | A | A | A | CVMNK |
| 39 | A | T | G |  | A | A | T |  | A | A | A | CVMNK |  | A | T | G |  | A | A | T |  | A | A | A | CVMNK |
| 40 | A | T | T |  | G | A | A |  | A | C | A | CVIET |  | A | T | T |  | G | A | A |  | A | C | A | CVIET |
| 41 | A | T | G |  | A | A | T |  | A | A | A | CVMNK |  | A | T | G |  | A | A | T |  | A | A | A | CVMNK |
| 42 | A | T | G |  | A | A | T |  | A | A | A | CVMNK |  | A | T | G |  | A | A | T |  | A | A | A | CVMNK |
| 43 | A | T | G |  | A | A | T |  | A | A | A | CVMNK |  | A | T | G |  | A | A | T |  | A | A | A | CVMNK |
| 44 | A | T | G |  | A | A | T |  | A | A | A | CVMNK |  | A | T | G |  | A | A | T |  | A | A | A | CVMNK |
| 45 | A | T | G |  | A | A | T |  | A | A | A | CVMNK |  | A | T | G |  | A | A | T |  | A | A | A | CVMNK |
| 46 | A | T | G |  | A | A | T |  | A | A | A | CVMNK |  | A | T | G |  | A | A | T |  | A | A | A | CVMNK |
| 47 | A | T | G |  | A | A | T |  | A | A | A | CVMNK |  | A | T | G |  | A | A | T |  | A | A | A | CVMNK |
| 48 | A | T | G |  | A | A | T |  | A | A | A | CVMNK |  | A | T | G |  | A | A | T |  | A | A | A | CVMNK |
| 49 | A | T | T |  | G | A | A |  | A | C | A | CVIET |  | A | T | T |  | G | A | A |  | A | C | A | CVIET |
| 50 | A | T | G |  | A | A | T |  | A | A | A | CVMNK |  | A | T | G |  | A | A | T |  | A | A | A | CVMNK |
| 51 | A | T | G |  | A | A | T |  | A | A | A | CVMNK |  | A | T | G |  | A | A | T |  | A | A | A | CVMNK |
| 52 | A | T | G |  | A | A | T |  | A | A | A | CVMNK |  | A | T | G |  | A | A | T |  | A | A | A | CVMNK |
| 53 | A | T | G |  | A | A | T |  | A | A | A | CVMNK |  | A | T | G |  | A | A | T |  | A | A | A | CVMNK |
| 54 | A | T | G |  | A | A | T |  | A | A | A | CVMNK |  | A | T | G |  | A | A | T |  | A | A | A | CVMNK |
| 55 | A | T | G |  | A | A | T |  | A | A | A | CVMNK |  | A | T | G |  | A | A | T |  | A | A | A | CVMNK |
| 56 | A | T | G |  | A | A | T |  | A | A | A | CVMNK |  | A | T | G |  | A | A | T |  | A | A | A | CVMNK |
| 57 | A | T | G |  | A | A | T |  | A | A | A | CVMNK |  | A | T | G |  | A | A | T |  | A | A | A | CVMNK |
| 58 | A | T | T |  | G | A | A |  | A | C | A | CVIET |  | A | T | T |  | G | A | A |  | A | C | A | CVIET |
| 59 | A | T | G |  | A | A | T |  | A | A | A | CVMNK |  | A | T | G |  | A | A | T |  | A | A | A | CVMNK |
| 60 | A | T | G |  | A | A | T |  | A | A | A | CVMNK |  | A | T | G |  | A | A | T |  | A | A | A | CVMNK |
| 61 | A | T | G |  | A | A | T |  | A | A | A | CVMNK |  | A | T | G |  | A | A | T |  | A | A | A | CVMNK |
| 62 | A | T | G |  | A | A | T |  | A | A | A | CVMNK |  | A | T | G |  | A | A | T |  | A | A | A | CVMNK |
| 63 | A | T | G |  | A | A | T |  | A | A | A | CVMNK |  | A | T | G |  | A | A | T |  | A | A | A | CVMNK |
| 64 | A | T | G |  | A | A | T |  | A | A | A | CVMNK |  | A | T | G |  | A | A | T |  | A | A | A | CVMNK |
| 65 | A | T | T |  | G | A | A |  | A | C | A | CVIET |  | A | T | T |  | G | A | A |  | A | C | A | CVIET |
| 66 | A | T | G |  | A | A | T |  | A | A | A | CVMNK |  | A | T | G |  | A | A | T |  | A | A | A | CVMNK |
| 67 | A | T | G |  | A | A | T |  | A | A | A | CVMNK |  | A | T | G |  | A | A | T |  | A | A | A | CVMNK |
| 68 | A | T | G |  | A | A | T |  | A | A | A | CVMNK |  | A | T | G |  | A | A | T |  | A | A | A | CVMNK |
| 69 | A | T | T |  | G | A | A |  | A | C | A | CVIET |  | A | T | T |  | G | A | A |  | A | C | A | CVIET |
| 70 | A | T | G |  | A | A | T |  | A | A | A | CVMNK |  | A | T | G |  | A | A | T |  | A | A | A | CVMNK |
| 71 | A | T | G |  | A | A | T |  | A | A | A | CVMNK |  | A | T | G |  | A | A | T |  | A | A | A | CVMNK |
| 72 | A | T | G |  | A | A | T |  | A | A | A | CVMNK |  | A | T | G |  | A | A | T |  | A | A | A | CVMNK |
| 73 | A | T | G |  | A | A | T |  | A | A | A | CVMNK |  | A | T | G |  | A | A | T |  | A | A | A | CVMNK |
| 74 | A | T | G |  | A | A | T |  | A | A | A | CVMNK |  | A | T | G |  | A | A | T |  | A | A | A | CVMNK |
| 75 | A | T | T |  | G | A | A |  | A | C | A | CVIET |  | A | T | T |  | G | A | A |  | A | C | A | CVIET |
| 76 | A | T | G |  | A | A | T |  | A | A | A | CVMNK |  | A | T | G |  | A | A | T |  | A | A | A | CVMNK |
| 77 | A | T | G |  | A | A | T |  | A | A | A | CVMNK |  | A | T | G |  | A | A | T |  | A | A | A | CVMNK |
| 78 | A | T | G |  | A | A | T |  | A | A | A | CVMNK |  | A | T | G |  | A | A | T |  | A | A | A | CVMNK |
| 79 | A | T | T |  | G | A | A |  | A | C | A | CVIET |  | A | T | T |  | G | A | A |  | A | C | A | CVIET |
| 80 | A | T | G |  | A | A | T |  | A | A | A | CVMNK |  | A | T | G |  | A | A | T |  | A | A | A | CVMNK |
| 81 | A | T | G |  | A | A | T |  | A | A | A | CVMNK |  | A | T | G |  | A | A | T |  | A | A | A | CVMNK |
| 82 | A | T | G/T |  | A/G | A | T/A |  | A | A/C | A | CV M/I N/E K/T |  | A | T | G/T |  | A/G | A | T/A |  | A | A/C | A | CV M/I N/E K/T |
| 83 | A | T | G |  | A | A | T |  | A | A | A | CVMNK |  | A | T | G |  | A | A | T |  | A | A | A | CVMNK |
| 84 | A | T | G |  | A | A | T |  | A | A | A | CVMNK |  | A | T | G |  | A | A | T |  | A | A | A | CVMNK |
| 85 | A | T | G |  | A | A | T |  | A | A | A | CVMNK |  | A | T | G |  | A | A | T |  | A | A | A | CVMNK |
